# Effect of oral calcium administration on metabolic status and uterine health of dairy cows with reduced postpartum rumination and eating time

**DOI:** 10.1186/s12917-021-02881-2

**Published:** 2021-04-29

**Authors:** Pablo Pinedo, Diego Manríquez, Nicolas Marotta, Giuliano Mongiello, Carlos Risco, Leen Leenaerts, Hans Bothe, Juan Velez

**Affiliations:** 1 Department of Animal Sciences, Colorado State University, Fort Collins, CO 80523-1171 USA; 2 Aurora Organic Farms, Platteville, CO 80651-9009 USA; 3 College of Veterinary Medicine, Oklahoma State University, Stillwater, OK 74078-2005 USA

**Keywords:** Rumination, Eating, Metabolic, Metritis

## Abstract

**Background:**

Hypocalcemia has detrimental effects on health and performance of dairy cows. As hypocalcemic cows show reduced feed intake, we hypothesized that cows with reduced combined rumination and eating time (CRET) may benefit from Ca supplementation. The objective was to evaluate the effect of postpartum oral Ca administration on metabolic status (Calcium [Ca], fatty acids [FA], and β-Hydroxybutyrate [BHB] serum concentrations) and incidence of puerperal metritis (PM) in dairy cows with reduced postpartum CRET. Cows in an organic-certified dairy, diagnosed with reduced CRET (< 489 min/d; *n* = 88) during the first day postpartum were assigned into 1 of 2 treatments: i) Calcium administration (CA; *n* = 45) that received 1 Ca oral capsule (Bovikalc bolus, Boehringer Ingelheim, St. Joseph, MO) containing CaCl2 and CaSO4 (43 g of Ca) once per day, for 3 consecutive days, starting at d 1 postpartum; and ii) Control (CON; *n* = 43) that did not receive oral Ca. A convenience group consisting of cows with CRET ≥489 min/d was used for comparison and did not receive oral Ca (NOR; *n* = 96).

**Results:**

At day 1 postpartum cows with reduced CRET had lower Ca serum concentrations (CA = 2.08 mmol/L; CON = 2.06 mmol/L) compared with NOR cows (2.17 mmol/L). Calcium concentrations at d 3, 5, and 12 postpartum were not different among the three groups. Serum FA concentrations at d 1, 3 and 5 postpartum were higher in both CA and CON cows compared with NOR. At d 12, only CA cows had higher FA concentrations than NOR cows. Serum BHB concentrations at d 3 were highest in CA, with no difference between CON and NOR. At d 5, BHB concentrations were higher in CA, followed by CON, and NOR. No effect was observed for Ca administration on incidence of PM and reproductive performance. CON cows had lower survival at 30 DIM (86.5%) than NOR cows (97.9%).

**Conclusions:**

The use of remote sensor technology identified cows with reduced rumination and eating time that had lower postpartum serum concentrations of calcium and altered metabolic status. However, oral calcium administration to cows with reduced CRET did not affect incidence of metabolic disorders nor reproductive health and subsequent pregnancy. Although survival at 30 days postpartum was lower for non-Ca supplemented cows, the identification of effective interventions in cows with reduced CRET requires further consideration.

## Background

Metabolic changes occurring at the time of calving represent a significant challenge for dairy cows [1]. The abrupt increase in nutrient requirements to support milk synthesis at a time when feed intake is depressed results in a shift from a positive to a negative energy balance that leads to fat mobilization and elevated fatty acids (FA) concentrations. Coincidently, peripartal synthesis and secretion of colostrum causes major losses of Ca in blood [2–4] and subclinical or clinical hypocalcemia may occur [5].

Hypocalcemic cows have decreased feed intake and rumination, increased plasma concentrations of cortisol and FA [6–8], limited numbers of neutrophils with phagocytic activity [4, 8, 9], and reduced concentrations of cytosolic Ca^2+^ in mononuclear cells [3]. The altered metabolic status and the impaired innate immune function of hypocalcemic cows leads to increased incidence of metritis and other diseases [4, 10], higher risk of culling, and reduced milk production and reproductive performance [11].

Oral Ca supplementation has been proposed as a complement to dietary strategies to manage hypocalcemia in dairy herds [12–14]. A recent study [15] reported that responses to oral Ca supplementation were conditional on parity and milk production level of cows. Oral Ca supplementation benefited reproduction in multiparous cows and milk yield in the cohort of multiparous cows of higher milk production potential, but Ca supplementation was detrimental to reproduction in primiparous cows. In addition to these contradictory effects, a blanket approach for oral calcium administration that includes normocalcemic cows might be economically questionable. Conversely, to optimize the effect of Ca supplementation, cows at risk of hypocalcemia should be targeted. However, as clinical signs of this condition are not evident, the identification of subclinically hypocalcemic cows remains a challenge.

The development and adoption of sensor technologies in the dairy industry has opened new capabilities for remote monitoring of health [16]. Due to the impact of reduced feed intake around calving on mineral and metabolic status, cows at risk of developing hypocalcemia could be identified by use of sensor technology capable of assessing rumination and eating behaviors [16].

Moreover, as low concentrations of Ca would exacerbate the decline in rumination and eating activity [7, 8, 10, 17, 18], these behavioral changes would likely be identified.

We hypothesized that early postpartum supplementation with oral Ca to cows identified with reduced feed intake and rumen activity would mitigate the negative effects of hypocalcemia, which includes a greater risk of metritis and metabolic disorders. Therefore, the objective was to evaluate the effect of postpartum oral Ca administration on metabolic status (total Calcium [Ca], fatty acids [FA], and β-Hydroxybutyrate [BHB] serum concentrations) and incidence of puerperal metritis (PM) in organic-certified dairy cows identified to have reduced rumination and eating time with the use of remote sensor technology. Other outcomes of interest included reproductive performance and survival up to 30 DIM.

## Results

### Descriptive statistics

The pre-enrollment analyses to determine a cut-off value for reduced postpartum combined rumination and eating time (CRET) demonstrated a significant association between CRET at d 1 postpartum and the occurrence of PM within the first 12 DIM. Healthy cows had CRET 193.2 (min/d) longer than PM cows on the day of calving (490.7 ± 8.7 vs. 297.5 ± 53 min/d, *P* = 0.0003). The area under the curve for the ROC analysis was 0.86, when a cut-off value of 489 min/d of CRET was considered in the model. The resulting sensitivity and specificity were 0.55 and 0.90, respectively. Therefore, cows in this study were classified according to their CRET during the first day postpartum as normal or having reduced CRET (CRET < 489 min/d).

**Fig. 1:**
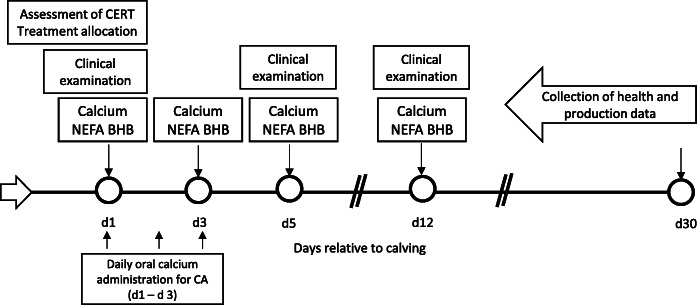
Study timeline in days relative to calving. At d 1, postpartum cows were classified according to their combined rumination and eating time (CRET) as normal or having reduced time (CRET < 489 min/d; *n =* 88). Cows diagnosed with reduced CRET were assigned randomly into 1 of 2 treatments: (1) Calcium supplemented (CA) received 1 Ca oral capsule (Bovikalc bolus, Boehringer Ingelheim, St. Joseph, MO) once per day, for 3 consecutive days; and (2) Control (CON) that did not receive any supplementation. A third group consisting of cows with normal levels of CRET (≥489 min/d) were enrolled as unaffected controls that remained untreated (NOR)

Based on this categorization, 184 lactating Holstein cows (57 primiparous and 127 multiparous) were enrolled in the study (calcium administration [CA] = 45; control [CON] = 43; unaffected controls [NOR] = 96) at d 1 postpartum (Fig. 1). One CON cow was euthanized due to toxic mastitis at 2 days postpartum, while 6 cows (CA = 1; CON = 3; NOR = 2) were sold within 12 days postpartum. Therefore, 178 and 177 cows were evaluated at d 5 and d 12, respectively.

**Fig. 2:**
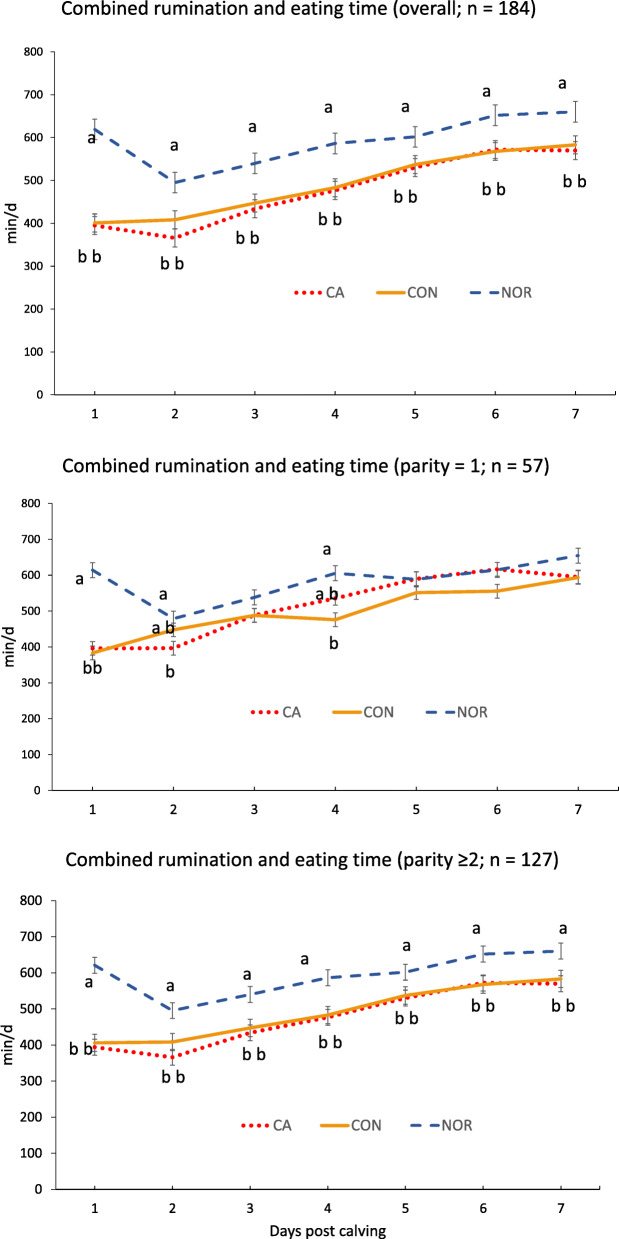
Least square means (± SEM) combined rumination and eating time from d 1 to d 7 after calving in the overall study population (top panel), in primiparous cows (middle panel) and in multiparous cows (bottom panel). Treatments: CA (red dotted line) = reduced combined rumination and eating time (CRET), received 1 Ca oral capsule (Bovikalc bolus, Boehringer Ingelheim, St. Joseph, MO) once per day, for 3 consecutive days; CON (orange solid line) = reduced CRET, did not receive any supplementation; NOR (blue dashed line) = normal CRET that did not receive any supplementation. Overall population, effects of treatment (*P* < 0.0001), d post calving (*P <* 0.0001), and treatment and d post calving interaction (*P* = 0.8). Primiparous, effects of treatment (*P =* 0.02), d post calving (*P* < 0.0001), and treatment and d post calving interaction (*P* = 0.7). Multiparous, effects of treatment (*P* = 0.0002) and d post calving (*P <* 0.0001), and treatment and d post calving interaction (*P =* 0.9). Different letters indicate significant differences (*P <* 0.01) between groups at specific sampling points

Average ± SD parity were 2.98 ± 1.68, 3.16 ± 1.67, and 2.35 ± 1.66 for CA, CON, and NOR, respectively (*P* = 0.08). Median (range) for lactation number in the overall population and in multiparous cows were 2 (1–8) and 3 (2–8), respectively. The proportions of primiparous cows were CA = 33%; CON = 26%, and NOR = 32% (*P* = 0.67). Average CRET at d 1 postpartum were 395.1 ± 80.9 min/d, 401.5 ± 80.0 min/d, and 619.1 ± 80.0 min/d for CA, CON, and NOR, respectively (*P* < 0.001). Average CRET by group from d 1 to d 7 postpartum are presented in Fig. 2. For multiparous cows, NOR had higher CRET values than CA and CON cows in all time points (*P* < 0.01). On the contrary, in primiparous cows there was no consistent trend to determine differences among the three treatments over time. Although CA and CON primiparous cows had reduced CRET at d 1, CA cows approached NOR’s rumination and eating time by d 3, while levels in CON were similar to NOR from d 2 to d 3 and from d 5 to d 7.

### Postpartum metabolic status

**Fig. 3:**
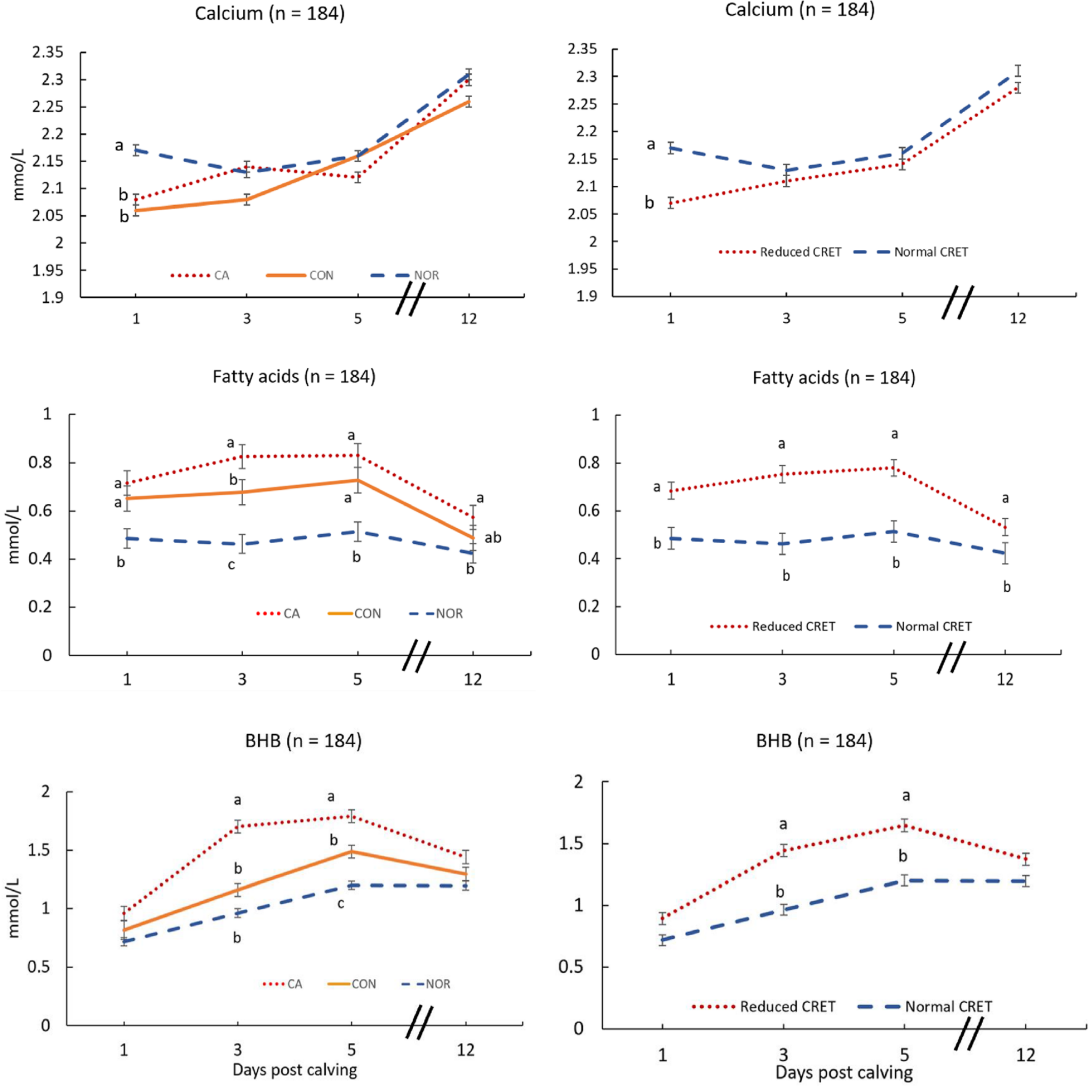
Least squares means (± SEM) from repeated measures analyses for postpartum Ca, FA, and BHB serum concentrations. Left panel: CA (red dotted line) = reduced combined rumination and eating time (CRET), received 1 Ca oral capsule (Bovikalc bolus, Boehringer Ingelheim, St. Joseph, MO) once per day, for 3 consecutive days; CON (orange solid line) = reduced CRET, did not receive any supplementation; NOR (blue dashed line) = normal CRET that did not receive any supplementation. Right panel: Normal CRET = NOR; Low CRET = CA and CON groups combined. Left panel: Calcium, effects of treatment (*P =* 0.03), parity (*P =* 0.002), d post calving (*P* < 0.001), and treatment and d post calving interaction (*P* = 0.017). Right panel: Calcium, effects of treatment (*P* = 0.02), parity (*P* = 0.014), d post calving (*P* < 0.001), and treatment and d post calving interaction (*P* = 0.106). Left panel: FA, effects of treatment (*P <* 0.0001), parity (*P* = 0.11), d post calving (*P <* 0.001), and treatment and d post calving interaction (*P* = 0.03). Right panel: FA, effects of treatment (*P <* 0.001), parity (*P* = 0.16), d post calving (*P <* 0.001), and treatment and d post calving interaction (*P* = 0.005). Left panel: BHB, effects of treatment (*P <* 0.001), parity (*P* = 0.0005), d post calving (*P <* 0.001), treatment and d post calving interaction (*P* = 0.007). Right panel: BHB, effects of treatment (*P <* 0.001), parity (*P* = 0.0014), d post calving (*P <* 0.001), treatment and d post calving interaction (*P* = 0.015). Different letters indicate significant differences (*P* < 0.05) between groups at specific sampling points. Different letters indicate significant differences (*P <* 0.05) between groups at specific sampling points. Diagonal lines indicate a break in the x-axis

All blood metabolites changed over time (*P* < 0.05), independent of Ca administration. Average ± SEM serum total Ca concentrations within 24 h postpartum were lower in CA (*P* < 0.001) and CON (*P* < 0.001), compared with NOR cows (CA = 2.08 ± 0.03 mmol/L; CON = 2.06 ± 0.03 mmol/L; NOR = 2.17 ± 0.02 mmol/L). The Ca concentrations in CA and CON cows were below the threshold considered to define sub clinical hypocalcemia (SCH) [4]. No significance for the interaction between treatment and parity was established (*P* = 0.41) and no differences in serum Ca concentrations were determined among groups from d 3 to d 12 (Fig. 3).

Average serum FA concentrations within 24 h postpartum were higher in CA (*P* = 0.002) and CON (*P* = 0.009), compared with NOR cows (CA = 0.71 ± 0.04 mmol/L; CON = 0.65 ± 0.04 mmol/L; and NOR = 0.48 ± 0.03 mmol/L), but no difference was established between CA and CON groups (*P* = 0.97). An effect for the interaction between treatment and parity was determined at 24 h postpartum (*P* = 0.02); only multiparous cows showed significant differences among treatments (CA = 0.80 ± 0.04 mmol/L vs. NOR = 0.48 ± 0.03 mmol/L [*P* < 0.001]; CON = 0.65 ± 0.04 mmol/L vs. NOR [*P* = 0.02]) (Fig. 3). No differences among groups were determined for average serum BHB concentrations within 24 h postpartum. However, higher BHB serum concentrations were observed in CA vs. CON (*P* = 0.04) and NOR (*P* < 0.001) at d 3 postpartum. At d 5 postpartum BHB serum concentrations were higher in CA vs. CON (*P* = 0.04) and NOR (*P* = 0.01) cows. Cows in CON had higher BHB values than NOR cows only at d 5 (*P* = 0.04; Fig. 3). Additional comparisons for Ca, FA, and BHB between reduced CRET and NOR cows (CON and CA combined) as are presented in Fig. 3.

Results for the association of treatment and CRET status with SCH, severe lipomobilization (SLM), and subclinical ketosis (SCK), while controlling for parity, can be found in Table 1. There was no effect of treatment or CRET status on the odds of SCH (*P* = 0.09 and *P* = 0.08, respectively), however there was an effect observed for both on the odds of SLM (*P* = 0.001 and *P* < 0.001, respectively) and SCK (*P* = 0.01 and *P* = 0.008, respectively). A tendency for lower SCH was determined in primiparous cows (*P* = 0.08) and the odds of SLM were more than double in primiparous than in multiparous cows (*P* = 0.03).

**Table 1:** Results from the multivariable logistic regression analyses for occurrence of metabolic diseases by treatment during the first 12 d after calving. Initial models included treatment (combined treatments), parity category (forced in the final models) and the interaction treatment by parity. As no significant effect for the interaction treatment by parity was established, this term was removed from the models

Condition	AOR^**a**^	95% CI^**b**^	***P***-value	Incidence		
**Hypocalcemia (Calcium ≤ 2.14 mmol/L)**						
				Treatment	+/n^d^	%
Treatment^c^			0.09			
CA vs. CON	0.44	0.15 - 1.31	0.14	CA	32/45	71.1
CA vs. NOR	1.28	0.59 - 2.79	0.53	CON	37/43	86.1
CON vs. NOR	2.89	1.10 - 7.65	0.03	NOR	63/96	65.6
Combined treatments						
Reduced CRET vs. NOR^e^	1.79	0.91 - 3.5	0.08			
**SLM (FA ≥ 0.6 mmol/L)**^**f**^						
Treatment			0.001			
CA vs. CON	0.98	0.35 - 2.79	0.97	CA	36/45	80.0
CA vs. NOR	3.95	1.71 - 9.15	0.001	CON	34/43	79.1
CON vs. NOR	4.02	1.71 - 9.43	0.001	NOR	49/96	51.0
Combined treatments						
Reduced CRET vs. NOR	3.98	2.05 - 7.76	< 0.001			
**SCK (BHB ≥ 1.0 mmol/L [d 1–4] and BHB ≥ 1.4 mmol/L [d 12])**^**g**^						
Treatment			0.01			
CA vs. CON	2.64	0.86 - 8.13	0.09	CA	39/45	86.6
CA vs. NOR	4.19	1.60 - 11.0	0.003	CON	32/43	74.4
CON vs. NOR	1.59	0.70 - 3.60	0.26	NOR	59/96	61.4
Combined treatments						
Reduced CRET vs. NOR	2.49	1.27 - 4.91	0.008			

^a^Adjusted odds ratios for the respective condition

^b^95% confidence interval

^c^Groups based on CRET at d 1 postpartum and allocated to two treatments: (1) Calcium supplemented (CA); and (2) Control (CON) that did not receive any supplementation. Unaffected controls (NOR; CRET ≥489 min/d) remained untreated

^d^Number of cases divided by treatment total

^e^Reduced CRET = Combined CA and CON

^f^SLM = Severe lipomobilization

^g^SKC = Subclinical ketosis

### Effect of treatment on puerperal metritis

The association of treatment and CRET status with prevalence of PM at d 5 and d 12, while controlling for parity, are shown in Table 2. The only significant differences were determined at d 12 for CA vs. NOR groups (*P* = 0.02) and for reduced CRET vs. NOR (*P* = 0.04). Although prevalence of PM at d 5 was lower in primiparous than in multiparous cows (8.8% vs. 14.3%), the effect of parity on PM at d 5 or at d 12 was not significant (*P* = 0.23 and *P* = 0.32, respectively). When both examination times where considered, cure rates at d 12 were 22.2, 33.3, and 55.6% for CA, CON, and NOR, respectively, with no difference by treatment group.

**Table 2:** Results from the multivariable logistic regression analyses for puerperal metritis diagnosis at d 5 and at d 12 after calving. Models included treatment (combined treatment), parity category (forced in the final models) and the interaction treatment by parity. As no significant effect for the interaction treatment by parity was established, this term was removed from the models

Condition	AOR^a^	95% CI^b^	*P*-value		Prevalence	
Puerperal metritis at d 5						
Treatment^c^				Treatment	+/n^d^	%
CA vs. CON	1.59	0.50 - 5.01	0.43	CA	9/44	20.5
CA vs. NOR	2.42	0.88 - 6.64	0.08	CON	6/40	15.0
CON vs. NOR	1.52	0.50 - 4.66	0.46	NOR	9/94	9.6
Combined treatments						
Reduced CRET vs. NOR^e^	1.97	0.81 - 4.80	0.13			
Puerperal metritis at d 12						
Treatment						
CA vs. CON	1.74	0.46 - 6.57	0.41	CA	7/44	15.9
CA vs. NOR	4.3	1.18 - 15.6	0.02	CON	4/39	10.3
CON vs. NOR	1.47	0.58 - 10.5	0.22	NOR	4/94	4.2
Combined treatments						
Reduced CRET vs. NOR	3.44	1.04 - 11.2	0.04			

^a^Adjusted odds ratios for the respective condition

^b^95% confidence interval

^c^Groups based on CRET at d 1 postpartum and allocated to two treatments: (1) Calcium supplemented (CA); and (2) Control (CON) that did not receive any supplementation. Unaffected controls (NOR; CRET ≥489 min/d) remained untreated

^d^Number of cases divided by treatment total

^e^Reduced CRET = Combined CA and CON

### Effect of treatment on pregnancy and survival

No significant differences by group were found for the proportions of cows pregnant at 200 days postpartum (CA = 39.8%; CON = 38.3%; and NOR = 47.3%; *P* = 0.2). Survival at 30 days postpartum were 93.3, 86.1, and 97.9% for CA, CON and NOR cows, respectively (*P* = 0.02). No significant differences in survival at 30 days postpartum were found for CA vs. CON and for CA vs. NOR, but the odds of survival for cows in CON were 0.16 (0.03–0.85; *P* = 0.03) times the odds of survival of NOR cows.

## Discussion

Due to the critical impact of reduced feed intake around calving on mineral and metabolic status, we anticipated that cows at higher risk of developing hypocalcemia and subsequent PM could be identified early after calving by use of remote sensor technology. Consequently, early postpartum supplementation with oral Ca to cows with reduced CRET would mitigate the effect of lower blood Ca concentrations, reducing the occurrence of puerperal metritis and improving subsequent fertility.

Supporting this concept, our preliminary analyses in the study farm indicated an association between reduced rumination and eating time at d 1 postpartum and occurrence of PM, as healthy cows spent 193 min/d longer than PM cows ruminating and eating during the day of calving. The completed ROC analysis resulted in sensitivity and specificity of 0.55 and 0.90 for the proposed cut-off value (CRET = 489 min/d). Relevant to the current study, this low sensitivity would result in failure to detect cows that were at risk of PM, but still had normal rumination and eating time. On the contrary, the resulting specificity suggests that the probability for incorrectly signaling a cow as being at high risk for PM was low. Although in this study the association between CRET at d 1 postpartum and the occurrence of PM was not replicated at d 5 postpartum, the odds of PM at d 12 were greater in cows with reduced CRET than in NOR cows.

The potential association between altered peripartal rumination and eating behavior and metabolic and reproductive disorders has been explored [4, 19, 20] and the adoption of health alerts provided by remote sensor technology is growing among farmers [21]. In a study by Liboreiro et al. [22], cows diagnosed with metritis had reduced rumination times during the postpartum period, while Stangaferro et al. [19] reported that the combination of activity and rumination was effective in identifying cows with severe cases of metritis or cows with metritis and another health disorder. In a study by Schirmann et al. [23], cows with metritis and SCK had lower dry matter intake during the precalving period and continued to eat less until d 20 postpartum, as compared with healthy cows. In agreement, Sahar et al. [24] reported that cows that spend less time eating during the first 90 min after fresh feed delivery in the prepartum period were at increased risk of developing SCK and metritis in the postpartum period. Interestingly, to the authors’ knowledge, the use of daily CRET to test these associations has not been widely explored. As both rumination and eating behaviors are affected before metritis presentation, it is plausible to suggest that this combination may be advantageous. In addition, establishing a fixed day for the assessment of CRET, as we propose here, would facilitate the logistics of diagnosis and treatment.

The concept of strategic Ca administration in specific subpopulations of cows has been proposed. Oetzel and Miller [14] determined that lame cows and higher producing cows responded favorably to oral Ca administration. In a more recent study, Martinez et al. [15] reported that responses to oral Ca supplementation depended on parity and milk production. Notably, while oral Ca supplementation benefited reproduction and increased milk yield in high producing multiparous cows, Ca supplementation had a negative effect in fertility of primiparous animals. Despite this indication of undesirable effects of Ca administration in primiparous cows, a proportion of first parity cows was included in the current study. Our rationale was to test the consistency of the detrimental effect of reduced CRET across lactations. We were also interested in establishing if oral Ca supplementation would be beneficial when administered to primiparous cows that could be at risk of hypocalcemia. Furthermore, as metritis was a relevant outcome of this study, primiparous cows that are at greater risk for this disorder were of special interest. Notably, in this study the interaction between treatment and parity was not significant and primiparous cows responded similarly to multiparous animals to treatment.

The main finding associated with parity category was that, contrary to multiparous, in primiparous cows there was no consistent trend in CRET values to determine differences among the three treatments over time (Fig. 2). A possible explanation for this lack of consistency is that primiparous cows moved to a new environment after calving and commingled with older multiparous cows could have a more erratic eating behavior following parturition. This condition, added to potential competition for eating space, could result in temporary reductions in CRET. However, following adjustment, some of these cows were able to return to greater levels of CRET soon after calving, narrowing the differences with NOR cows.

As anticipated, in the current study the two groups with reduced CRET at d 1 postpartum had lower serum Ca concentrations (SCH), while serum FA concentrations were higher compared with NOR cows. In agreement with this finding, a recent study by Goff et al. [18] indicated that normocalcemic cows spent more time ruminating on the first day after calving than subclinically hypocalcemic cows or cows with milk fever. Moreover, plasma Ca concentration at 12 h postpartum was also correlated with rumination rate on the second day after calving, even though blood Ca concentrations had increased in most cows by d 2.

The link between postpartum rumination and metabolic disorders was explored by Soriani et al. [25] that indicated a negative correlation between rumination time and BHB. In that study, cows with shorter rumination time maintained greater BHB levels up to 35 DIM, suggesting prolonged SLM. Similarly, Stangaferro et al. [26] described higher plasma concentrations of FA and BHB and lower concentrations of Ca in postpartum cows with altered values of an index combining rumination time and physical activity. Nonetheless, whether rumination and activity were lower because of the underlying condition affecting the cow and its effect on feeding behavior, or because of the altered levels of the markers measured could not be determined in these studies.

In a recent study, Pérez-Báez et al. [27] demonstrated that cows that developed ketosis had lesser DMI relative to their BW and lesser energy balance on d − 5, − 3, − 2, and − 1 than cows without ketosis. After calving, cows with ketosis maintained decreased DMI relative to BW accompanied by lesser energy balance and greater energy-corrected milk. Overall, these findings agree with greater FA and BHB concentrations reported in our study in reduced CRET cows (Fig. 3) that had to increase their mobilization of energy reserves, disrupting their metabolic status.

As anticipated in the present study, Ca was lower in reduced CRET cows sampled at calving. However, samples between d 3 and d 12 postpartum indicated no differences in concentrations of Ca between the supplemented and the untreated reduced CRET group and between these two groups and NOR cows (Fig. 3). A transient increase in serum Ca concentration was observed at d 3 in CA cows, but the magnitude of the difference relative to CON cows was not large enough to indicate significance and serum Ca concentrations returned to lower values at d 5.

Oetzel and Miller [14] reported that administration of oral Ca did not improve ionized Ca concentrations determined 20 h after bolus administration, while Melendez et al. [28] concluded that oral Ca supplements resulted in no effect on plasma concentrations of Ca measured every 24 h. Considering these reports, a sustained recovery on Ca levels due to the direct effect of oral CA administration in CA cows was not anticipated in our study. However, we projected that rumination and eating time would increase faster in CA, allowing for a more rapid recovery from low Ca levels, as compared to CON cows. As shown in Fig. 2, the recovery in rumination and eating time in CA cows was not faster than that of CON cows and both CA and CON multiparous cows were consistently under the NOR group.

Considering the potential inability of sustaining adequate concentrations of serum Ca after administration, the dose of Ca used in the current study (43 g/d of Ca for 3 d) was higher than that of two previous reports [14, 29], where 1 or 2 oral Ca boluses containing 43 g of Ca were administered after calving. Although it is plausible to infer that the dose of Ca supplementation used in these studies was insufficient, higher concentrations of Ca supplementation (86 g/d of Ca on d 0 and 1 postpartum, followed by 43 g/d on d 2, 3, and 4 postpartum) tested by Martinez et al. [15] reduced SCH but increased the incidence of metritis and negatively affected reproduction in primiparous cows considered to be at lower risk of metritis. In our study, a clear assessment of the impact of treatment on serum Ca concentrations following calving and the duration of this effect in CA cows was not possible due to the lack of post treatment samples on d 1 and d 2, which is a limitation of the current study.

Although cows at higher risk of hypocalcemia and suboptimal metabolic and health status were identified by use of CRET, the calcium treatment intervention was not effective on the outcomes evaluated. Relevant to our main objective, no difference was established in SCH incidence between CA and CON cows (Table 1), likely due to an insufficient impact of Ca administration on CRET (Fig. 2). Nonetheless, considering that the incidence of SCH in CA was close to the midpoint between CON and NOR cows, the limited sample size could also have a role on this assessment. Moreover, when CA and CON cows were compared with NOR, only CON cows had higher incidence of SCH.

As reported by Grummer et al. [1], limited feed intake from reduced CRET had a consistent effect on the incidences of overall SLM and SCK when all the sampling points were combined. In our study, cows with reduced CRET likely experienced a more pronounced negative energy balance that resulted in higher incidences of overall SLM in CA and CON compared with NOR cows, as it was also the case for SCK in CA vs. NOR. Overall, incidences of SCH, SLM, and SCK were higher than those reported in other studies [30]. This could reflect the specific dynamics of the study farm, as well as the criteria used in this study, where at least one positive result within the four consecutive samples was enough to consider a cow affected. The use of this criteria results in increments in sensitivity, but an increase in the proportion of false positives is also expected. Nonetheless, this discrepancies with values in other populations suggest caution regarding the external validity of these findings.

The lack of effect of Ca supplementation on serum concentrations of Ca, FA, or BHB in the current study agrees with data reported by Melendez et al. [28], that also administered oral Ca to multiparous cows with no effect. Moreover, FA and BHB were higher in the Ca supplemented group than in CON at d 3 and at d 3 and d 5, respectively (Fig. 3). Interestingly, primiparous cows had higher incidence of SLM, which disagrees with data presented by McArt et al. [31].

In our study, prepartum primiparous and multiparous cows were comingled, which could play a role on the high levels of SLM in primiparous cows, as competition for eating space could arise, affecting the feed intake after calving even in NOR cows (Fig. 2).

In support of this idea, only multiparous cows showed treatment group differences for SLM incidence within 24 h postpartum. However, an explanation for this finding would be conjectural due to the information available in this study.

In the current study cows with reduced CRET during the first day postpartum had lower serum Ca concentrations and altered metabolic status. However, in disagreement with previous reports [19, 22], no differences in the occurrence of PM at d 5 among treatments or between reduced CRET and NOR were found. At d 12, though, the odds of PM were more than four times higher for CA than for NOR and, when CA and CON groups were combined, the odds of PM were more than three times higher for reduced CRET cows than for NOR cows. Therefore, the suggested association between reduced CRET and PM was only partially replicated in this study.

When cure rates at d 12 were compared, no significant differences were found for any of the group comparisons. This lack of response to Ca administration agrees with the study by Pinedo et al. [32], were supplementing oral calcium at the time of PM treatment had no effect on clinical cure, defined as the combination of absence of fever and (vaginal discharge score) VDS < 4 at d 6 and 12 post treatment.

The association between SCH and reproductive disorders has been extensively reported [33–35] and may be in part related to the lack of adequate ionized Ca concentrations required for uterine smooth muscle contractions [7], together with limited supply of glucose and ionized Ca for competent immune function. Interestingly, cows diagnosed with PM at d 5 had lower concentrations of serum Ca compared with healthy cows (2.06 mmol/L vs. 2.18 mmol/L), which agrees with the study by Pinedo et al. [32] where cows affected with PM had lower calcium concentrations at diagnosis (1.57 mmol/L) compared with matched control healthy cows (2.10 mmol/L) and the prevalence of serum Ca < 2.15 mmol/L was 90 and 49% for PM and healthy groups, respectively.

Although not significant, the lower prevalence of PM at d 5 in primiparous compared to multiparous cows was an unexpected finding that contradicts previous reports indicating a higher risk for metritis in primiparous cows associated with a higher need for calving assistance [36]. A possible explanation for this contradictory finding may be the study exclusion criterium for cows with severe dystocia, which is the main reason for higher levels of metritis in primiparous cows.

The required sample size for this study was calculated anticipating a beneficial effect of treatment on metabolic status. Specifically, a difference in FA serum concentrations at d 5 postpartum in CA vs. CON that was considered. The rationale was that a deeper negative energy balance in cows with reduced CRET would result in elevated FA. Our expectation was that oral Ca supplementation to CA cows would increase rumination and eating time similar to NOR cows. Subsequently, increased DMI in CA cows would favor a decrease in their FA levels. However, as presented in Fig. 2, postpartum CRET were not substantially different between CA and CON cows, which likely affected the significance of the differences in the other study outcomes. In addition, a limitation of the current study is that for binary outcomes, such as presentation of PM and other health disorders, considerable differences among treatments would be needed to determine statistical significance. As an example, although PM prevalence at d 5 was 20.5% vs. 9.6% in CA and NOR, due to power limitations, the magnitude of this difference was not large enough to establish statistical significance. On the contrary, at d 12 a significant difference on PM prevalence was established between CA (15.9%) and NOR (4.2%) groups (Table 2).

Serum Ca concentrations in CA and CON were lower than those of NOR at d 1; however, Ca concentrations in these two groups increased and became similar to NOR by d 3 in CA and d 5 in CON cows. In a recent report by Neves et al. [37] plasma Ca concentration assessed at 1 postpartum was not associated with the risk of metritis in primiparous cows. However, an association was determined at 2, 3, and 4 days postpartum. An increased risk of metritis was established at 2 DIM for 2nd lactation animals and at 4 DIM for 3rd and higher lactation. In a second study by that group [38], immediate postpartum plasma Ca concentrations (within 12 h of parturition) were poor predictors of metritis. In the study presented here, CRET cows had average Ca serum concentrations lower than NOR cows only at 1 DIM. In agreement with the findings presented by Neves et al. [37, 38], the transient nature of lower Ca concentrations in CA and CON could be an explanation for the lack of a clear association between treatment and PM in this study.

Subclinical hypocalcemia is a well-described risk factor for reduced reproductive performance and increased risk of culling. Cows with SCH had reduced estrous cyclicity, pregnancy at first AI, and extended median days to pregnancy [4, 11]. The exclusive use of natural service in the study farm provided a unique opportunity for testing fertility differences in the treatments without the potential effect of variable estrus detection efficiency and compliance in estrus synchronization protocols. In addition, all cows were exposed to bulls for natural service at the same day postpartum and therefore had similar days postpartum for breeding. Possible factors explaining the low percentage of cows pregnant at 200 DIM could be that breeding took place in summer, as the study cows calved at the end of spring (April to July). In addition, due to prohibitions in the use of hormones in organic certified systems, none of the cows were subjected to any type of estrus or ovulation synchronization protocol.

Our results show that Ca administration had no effect in pregnancy at 200 DIM, although there was a difference in the LSM of 7.5 and 9% points for NOR vs. CA and CON cows, respectively. The lack of differences in pregnancy between CON and Ca supplemented cows is in partial agreement with previous research on PM cows supplemented with Ca after diagnosis [39]. Although in that study Ca supplementation to PM cows increased the proportion of cows inseminated by 150 DIM, no effect on pregnancy was determined. Interestingly, in a study by Martinez at al [15]., Ca supplementation to primiparous cows resulted in reduced P/AI at first and all artificial inseminations, extended median days to pregnancy, and smaller proportion of pregnant cows. However, Ca supplementation to multiparous cows improved P/AI at the first and all artificial inseminations, resulted shorter days to pregnancy and increased proportion of pregnant cows.

In the current study there was lower survival at 30 DIM in CON cows compared with NOR cows (86.5% vs. 97.9%). Although survival was higher in calcium supplemented cows (93.3%) than in CON cows, this difference was not significant. Interestingly, PM cows did not have lower survival than healthy cows, which contrasts with a previous report where survival at 30 DIM were 72.6 and 84.1% for two groups of cows with PM, compared with 98% in healthy matched controls [32].

Due to the organic-certified status of the study dairy, health and reproductive management practices may affect the external validity of the current findings [40]. The prohibition on the use of antibiotics limits the treatment efficacy of infectious disease such as PM, which may have affected clinical cure rates for this disorder. Moreover, the study cows were managed under natural service without any use of artificial hormones, which is also a distinct scenario.

In consequence, the moderated sample size and the specific health and reproductive management practices to be compliant with the organic certification in the study farm suggest caution regarding the external validity of our findings. Further research with a larger population under multiple management settings should be conducted to confirm the results reported in this study.

## Conclusions

The use of remote sensor technology identified cows with reduced rumination and eating time that had lower postpartum serum concentrations of calcium and altered metabolic status. However, oral calcium administration to cows with reduced CRET did not affect incidence of metabolic disorders nor reproductive health and subsequent pregnancy. Although survival at 30 days postpartum was lower for non-Ca supplemented cows, the identification of effective interventions in cows with reduced CRET requires further consideration.

## Methods

### Study population

The study population consisted of Holstein cows calving from April 23 to July 14, 2019 in a single certified organic dairy farm (1230 milking cows) located in Northern Colorado, USA. All cows were housed in dry lots, with access to shade and shelter that included straw bedding. Prepartum primiparous and multiparous cows were comingled. Cows were milked twice daily, and milk yield was recorded and analyzed for fat, protein, and SCS monthly for individual cows. The rolling herd average milk production was 8100 kg. Cows were fed a TMR twice a day to meet or exceed the nutritional requirements for a lactating Holstein cow producing 30 kg/d of milk with 3.5% fat and 3.1% true protein [41]. During the grazing season (April to September), cows had access to pasture and grazing provided at least 30% of the DMI of the total ration. Grazing management consisted of rotational grazing in pastures based on perennial forages, alfalfa, Italian rye grass, oat rye grass, and teff grass. The TMR was based on corn silage (5 to 7%), wheat silage (17 to 19%), grain mix containing soybean, soy hulls, corn, wheat, and minerals and vitamins (38 to 41%), sorghum silage (5 to 7%), alfalfa hay (2%), grass hay (0 to 1.5%), and pasture grazing (estimates as 30 to 38%).

Nongrazing diets for lactating cows were based on corn silage (14–17.5%); wheat silage (13–20%); grain mix containing soybean, soy hulls, corn wheat, mineral, and vitamins (47.5–50.5%); sorghum silage (3.0–4.5%); alfalfa hay (12–16%); and grass hay (0–3%).

Through the study period prepartum diet was based on corn silage (10.5 to 16.0%, DM basis), wheat silage (10.5 to 16.6%), alfalfa hay (32 to 41%), grass hay (14.2 to 20%), grain mix (12.6 to 19.2%), and mineral and vitamin mix (3.8%). Anionic salts were included into the ration (dietary cation anion difference = − 100 mEq/kg).

Cows enrolled in the study were subjected to a reproductive program based on natural service. Cows were moved to the bull pens at 30 DIM and pregnancy diagnosis was performed by transrectal palpation of the uterus and its content on a weekly basis, starting at 70 DIM. Cows diagnosed non pregnant were checked every 2 weeks. Due to prohibitions in the use of hormones in organic certified systems, none of the cows were subjected to any type of estrus or ovulation synchronization protocol.

### Case definitions and treatment allocation

All cows that calved during the study period, starting on April 23, 2019, were eligible for enrollment. Cows that had calving related problems (severe dystocia, cesarean section, fetotomy, stillbirth, twins, or uterine prolapse) were excluded from enrollment. Individual CRET (min/d) was determined using previously validated accelerometers (CowManager SensOor, Agis Automatisering BV, Harmelen, the Netherlands) tagged in the left ear [42, 43]. The accelerometers were designed to differentiate spatial movements of the ear being associated to rumination, eating, and activity (walking-running) and provided a reliable approximation of the time cows spent ruminating and eating. As this was a regular management practice in the study farm, all the cows were affixed with the accelerometer as heifers.

To identify an adequate cut-off maximizing CRET sensitivity and specificity to determine the risk for PM, a retrospective analysis was completed considering 480 cows housed in the study dairy. The analysis was performed using cow records prior to the start of the study. These data consisted of a single cohort of cows (primiparous = 152; multiparous = 328) with lactations started between October 2018 and March 2019 (mean ± SD lactation number = 2.6 ± 1.6). The association between CRET at d 1 postpartum and the occurrence of PM within the first 12 DIM was tested by ROC analysis, maximizing the area under the curve and the combined values for sensitivity and specificity. The CRET value selected for cut-off in this population was 489 min/d.

Consequently, for the current study, cows were classified according to their CRET during the first day postpartum as normal or having reduced CRET (CRET < 489 min/d). Cows diagnosed with reduced CRET were blocked by parity (1; ≥2) for treatment allocation. The assignment of the first cow in each block (primiparous/multiparous) was determined by tossing a coin, followed by a sequential allocation into 1 of 2 treatments: (1) Calcium administration (CA; *n* = 45) received 1 Ca oral capsule (Bovikalc bolus, Boehringer Ingelheim, St. Joseph, MO) once per day, for 3 consecutive days; and (2) Control (CON; *n* = 43) did not receive calcium administration. A convenience group consisting of cows with CRET ≥489 min/d was enrolled as unaffected controls that remained untreated (NOR; *n* = 96).

Calcium oral capsules contained CaCl2 and CaSO4 (43 g of Ca). The total dose for the 3 days treatment regimen was 129 g of Ca (43 g/d) per cow. The total amount of Ca administered for the complete treatment was greater than that reported by Oetzel and Miller [14] and Sampson et al. [29] that supplemented cows with 1 or 2 oral Ca boluses (Bovikalc bolus, Boehringer Ingelheim, St. Joseph, MO) after calving. None of the study cows in the three groups received any oral or parenteral Ca other than the treatments described above and the Ca contained in the diet.

### Postpartum health-monitoring program, outcomes of interest, and independent variables

All postpartum cows were monitored daily for general health from calving to 12 DIM. Monitoring was performed between 0600 and 0800 h, following the AM milking. Main parameters considered were appetite, appearance (depressed, dehydrated), and characteristics of vaginal discharge (color, consistency, and odor). Cows that appeared abnormal on any of these parameters were submitted for clinical examination by a veterinarian.

Enrolled cows fitting our inclusion criteria had complete clinical examination performed by a veterinarian blinded to allocation groups and the nature of treatments at enrollment and at d 5 and 12 postpartum. Vaginal discharge was evaluated using the Metricheck device (Metricheck, SimcroTech, Hamilton, New Zealand) and scored (VDS) according to Chenault et al. [44] as follows: 0 = no discharge observed; 1 = not fetid, normal lochia (viscous; red, brown, or clear); 2 = not fetid; thick mucus; cloudy, clearing, or clear; 3 = not fetid; may be purulent, mucopurulent, or chocolate brown; or 4 = fetid; may be red or pink to chocolate brown; thin, serous, and watery; with or without pieces of necrotic tissue. Rectal temperature (RT) was measured using an electronic thermometer (GLA Agricultural Electronics, San Luis Obispo, CA). Cows with PM were defined as having an abnormally enlarged uterus with VDS of 4, signs of systemic illness (dullness, or other signs of toxemia) and fever (RT ≥39.5 °C) [45]. Cows with PM were treated according to the study farm health protocols. Treatments were applied every other day for a total of 3 consecutive treatments as follows: Cows received 3.75 mL of Optimum UterFlush (Van Beek Natural Science, USA, containing yucca extract, cinnameldahyde, thymol, and a proprietary blend of carvacrol [4-isopropyl-2-methylphenol]) diluted in 117 mL of distilled water intrauterus. In addition, all PM treated cows received hypertonic saline solution (500 mL of 7.2% i.v.), dextrose (500 mL of 50% i.v.), and oral aspirin [5 boluses (15.6 g of acetylsalicylic acid/bolus)/d for 3 d]. According to organic certification, no antibiotic therapies were used in the study farm. Clinical cure in cows treated for PM at d 5 was defined as the combination of absence of fever and VDS < 4 at d 12 examination [44].

All the enrolled cows had whole-blood samples collected from the middle coccygeal vein using 20-gauge needles into sterile glass Vacutainer tubes without anticoagulant (BD Vacutainer Precision Glide, Becton Dickinson, Franklin Lakes, NJ). All samples were collected following the AM milking, between 0600 and 0800 h, before oral Ca administration. Samples were refrigerated and centrifuged at 1500×*g* for 15 min at 18 °C within 2 h of collection. Serum was harvested and stored frozen at -80 °C for subsequent analyses at the Veterinary Diagnostic Laboratories at Colorado State University (Fort Collins, CO). Sampling points included 24 h after calving and d 3, 5, and 12 postpartum and the analyses included total Ca, FA, and BHB determination, as this is the period when cows typically experience reduced blood Ca concentrations [3, 29] and negative energy balance [46] (Fig. 1).

Subclinical hypocalcemia was considered when serum Ca concentration ≤ 2.14 mmol/L [4]. Lactating cows were under severe lipomobilization (SLM) if serum FA concentration ≥ 0.6 mmol/L [46, 47]. Subclinical ketosis (SCK) was defined as serum BHB ≥1.0 mmol/L for sampling points at d 1, 3, and 5 and serum BHB ≥1.4 mmol/L for d 12 [11, 46, 48, 49]. Overall SCH, severe negative energy balance, or SCK status were determined for each study cow considering all the blood sample collection points (1 = at least one positive result at d 1, 3, 5, 12 postpartum).

Pregnancy diagnosis outcomes and dates of removal from the herd and the recorded reason for exit were also exported from PCDART® (Dairy Records Management Systems, Raleigh, NC), for assessment of reproductive performance (percentage of cows pregnant at 200 DIM) and survival at 30 DIM.

### Statistical analysis

The experimental design was a randomized block design with cows with reduced CRET blocked by parity (1; ≥2) and assigned randomly into 1 of 2 treatments. Sample size calculations were performed using the data analysis application SAS Power and Sample Size (release 9.4; SAS, Inst. Inc., Cary, NC). The sample size was calculated anticipating the potential impact of oral Ca supplementation in reduced CRET cows on metabolic status. Considering the reported association between elevated serum concentrations of FA and health and fertility outcomes [47, 50], this metabolite was used in the calculations. Previously reported FA values were considered in the calculations [5, 8, 15]. The goal was to detect a difference of 0.09 mmol/L in FA serum concentrations at d 5 postpartum in CA (0.71 mmol/L) vs. CON (0.80 mmol/L) cows. Considering SD = 0.17 mm/L and power = 80% and confidence = 95%, the number of cows required to determine a significant difference between the 2 groups was estimated in 44 cows per group.

The cut-off for reduced CRET was determined before enrollment from a preliminary dataset within the study farm, including individual general data, rumination and eating times, as well as PM events. The association between CRET at d 1 postpartum and the occurrence of PM within the first 12 DIM was tested by ROC analysis. Receiving operating characteristic curves were created using the ROC option in PROC LOGISTIC. This option generated classification tables with probability levels containing sensitivity and specificity values. Multiple cut-off values were tested maximizing the area under the curve and the combined values for sensitivity and specificity. The CRET value selected for cut-off in this population was 489 min/d.

Rumination and eating times were collected as min/h from calving until 30 DIM. Individual CRET were summarized into min/d using the PROC SQL procedure. For model building, individual explanatory variables were initially tested in univariable models. Explanatory variables associated with each outcome at *P* < 0.25 in the univariable analyses were initially included in the multivariable models. The multivariable models also included two-way interactions between all the predictor variables, which were kept in the final models if *P* ≤ 0.10. A backward stepwise elimination procedure was used. At each phase, the explanatory variable with largest *P*-value was removed if *P* > 0.10. At each stage, model fit was assessed using the Akaike’s information criterion.

Daily LSM for CRET were estimated for PM and healthy cows using PROC MIXED for repeated measures. This model considered PM status and DIM, and an interaction term between PM status and DIM, with cow nested within treatment, included as a random effect. The association between increments of CRET at calving day and the likelihood of PM was assessed using PROC LOGISTIC including the classification table option for determining the AUC and the probability levels to calculate the cut-offs of CRET that maximized sensitivity and specificity. All data were exported to Microsoft Excel (Microsoft Corp., Redmond, WA), where the information was organized for further analysis using SAS. The appropriate randomization of cows at the time of enrollment for discrete outcomes (parity number and proportion of primiparous cows) was analyzed by the Chi-square test (PROC FREQ). Logistic regression (PROC GLIMMIX) was used for the analysis of PM binary outcome variables (SCH, SLM, SCK) with results reported as adjusted odds ratios. Continuous variables (metabolites concentrations) were evaluated by ANOVA (PROC MIXED). For both types of variables, treatment (combined treatments) and parity category (1; ≥2), considered as a potential confounder, were forced in the final models. The initial models included parity category by treatment interaction as covariable. This effect was considered for inclusion at *P* < 0.10. Repeated measures analyses using the MIXED procedure, assuming an exchangeable covariance structure, were used to test the effects of treatment, parity category, and blood sampling day relative to calving (time of sampling) for each blood metabolite (total Ca, FA, BHB). Time of sampling was included as a repeated measure with cow nested within treatment considered as a random effect. The interaction effects treatment by parity category and treatment by sampling day were included in the initial models, but only the interaction treatment by sampling day remained in the final models. The Tukey multiple differences test was used to compare treatments by day where appropriate. The analyses tested differences among treatments (CA vs. CON. Vs. NOR), as well as between reduced CRET (CA + CON) and normal CRET (NOR). All results are expressed as adjusted odds ratios and least squares means with their respective 95% confidence intervals or standard errors. Significance and tendency levels were declared at *P* < 0.05 and *P* < 0.1, respectively.

## Data Availability

Data sets generated from this study are available upon request to the corresponding author.

## Abbreviations

BHB: β-Hydroxybutyrate
CA: Calcium administration group
CON: Control group
CRET: Combined rumination and eating time
FA: Fatty acids
LSM: Least squares means
MP: Multiparous cows
NEB: Negative energy balance
NOR: Normal unaffected controls
PM: Puerperal metritis
SCH: Subclinical hypocalcemia
SCK: Subclinical ketosis
SLM: Severe lipomobilization
VDS: Vaginal discharge score
